# Thioredoxin Profiling of Multiple Thioredoxin-Like Proteins in *Staphylococcus aureus*

**DOI:** 10.3389/fmicb.2018.02385

**Published:** 2018-10-15

**Authors:** Hui Peng, Yixiang Zhang, Jonathan C. Trinidad, David P. Giedroc

**Affiliations:** ^1^Department of Chemistry, Indiana University Bloomington, Bloomington, IN, United States; ^2^Biochemistry Graduate Program, Indiana University Bloomington, Bloomington, IN, United States; ^3^Laboratory for Biological Mass Spectrometry, Department of Chemistry, Indiana University Bloomington, Bloomington, IN, United States; ^4^Department of Molecular and Cellular Biochemistry, Indiana University Bloomington, Bloomington, IN, United States

**Keywords:** thioredoxin-like protein, thioredoxin profiling, hydrogen sulfide, protein persulfide reduction, pyruvate kinase

## Abstract

Hydrogen sulfide (H_2_S) is thought to signal through protein *S*-sulfuration (persulfidation; *S*-sulfhydration) in both mammalian systems and bacteria. We previously profiled proteome *S*-sulfuration in *Staphylococcus aureus* (*S. aureus*) and identified two thioredoxin-like proteins, designated TrxP and TrxQ, that were capable of reducing protein persulfides as a potential regulatory mechanism. In this study, we further characterize TrxP, TrxQ and the canonical thioredoxin, TrxA, by identifying candidate protein substrates in *S. aureus* cells using a mechanism-based profiling assay where we trap mixed disulfides that exist between the attacking cysteine of a FLAG-tagged Trx and a persulfidated cysteine on the candidate substrate protein in cells. Largely non-overlapping sets of four, 32 and three candidate cellular substrates were detected for TrxA, TrxP, and TrxQ, respectively, many of which were previously identified as global proteome *S*-sulfuration targets including for example, pyruvate kinase, PykA. Both TrxA (*k*_cat_ = 0.13 s^-1^) and TrxP (*k*_cat_ = 0.088 s^-1^) are capable of reducing protein persulfides on PykA, a model substrate detected as a candidate substrate of TrxP; in contrast, TrxQ shows lower activity (*k*_cat_ = 0.015 s^-1^). This work reveals that protein *S*-sulfuration, central to H_2_S and reactive sulfur species (RSS) signaling, may impact cellular activities and appears to be regulated in *S. aureus* largely by TrxP under conditions of sulfide stress.

## Introduction

Hydrogen sulfide (H_2_S) has long been studied as an environmental toxin, but is now generally recognized as the third “gasotransmitter,” alongside nitric oxide (NO), and carbon monoxide (CO), which signal at physiologically relevant concentrations in the vascular and nervous systems (Wang, 2002). H_2_S and its downstream more oxidized sulfur-containing molecules, collectively known as reactive sulfur species (RSS), signal via protein *S*-sulfuration (also known as persulfidation or *S*-sulfhydration) in both mammalian cells and in bacteria (Mustafa et al., 2009; Ida et al., 2014). For example, H_2_S causes hyperpolarization and vasorelaxation by inducing *S*-sulfuration of the ATP-sensitive potassium channel, which in turn activates its transport activity (Zhao et al., 2001). In contrast to activation of enzyme activity, *S*-sulfuration can also be inhibitory, often by targeting an active site cysteine, e.g., in PTP1B and glyceraldehyde 3-phosphate dehydrogenase (GAPDH) (Krishnan et al., 2011; Hildebrandt et al., 2015; Peng et al., 2017b). Given the ability of *S*-sulfuration to impact protein activities either directly or indirectly, it seems possible that this posttranslational modification (PTM) may be dynamically regulated to maintain cellular function.

**FIGURE 1:**
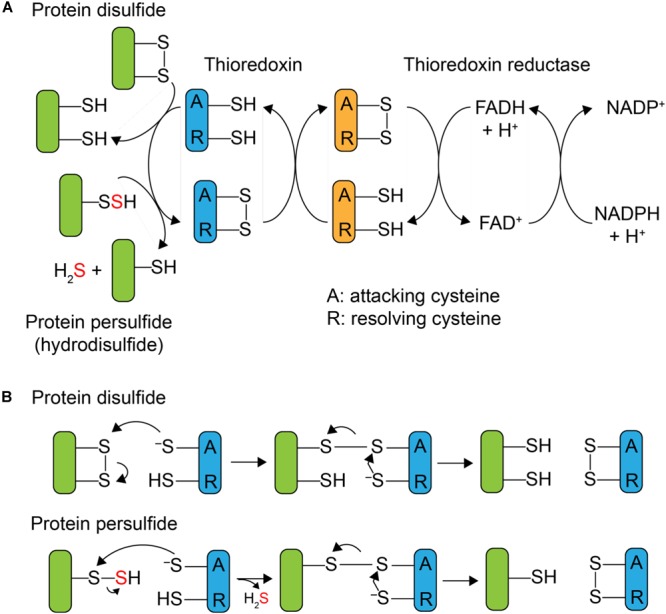
Mechanism of Trx-catalyzed disulfide vs. persulfide reduction. **(A)** General mechanism of the 2 e^-^ reduction of a substrate protein disulfide vs. protein persulfide (shaded *green*) by a thioredoxin or Trx-like protein (shaded *blue*), with the attacking (labeled A) and resolving (label R) cysteines indicated. The attacking and resolving Cys on thioredoxin reductase (shaded o*range)* reduce Trx as indicated. **(B)** Parallel mechanisms of the 2 e^-^ reductions of a protein disulfide vs. persulfide (*S*-sulfurated Cys; a hydrodisulfide) by Trx, each of which form a common mixed Trx-substrate disulfide intermediate (*middle*) that will not yield products when the resolving Cys is mutated to a serine; this is basis of the Trx profiling experiments presented here (see **Figure 3**).

Although protein *S*-sulfuration is a reversible PTM, it remains unclear whether protein persulfide reduction, i.e., depersulfidation, occurs spontaneously by reducing reagent, or enzymatically, as in phosphorylation and *S*-nitrosation (Lu et al., 2013). In general, thioredoxin (Trx) and its partner reductase, thioredoxin reductase, mediate the two-electron reduction of a disulfide bond and regenerate reduced Trx. After it was shown that Trx was capable of reducing both protein nitrosothiols, RSNO, with the release of HSNO (Benhar et al., 2008) and sulfenylated cysteines, RSOH (Lo Conte and Carroll, 2013), mammalian Trx was subsequently reported to reduce protein persulfides as well, with the presumed release of H_2_S and the reduced thiol (Dóka et al., 2016; Ju et al., 2016; Wedmann et al., 2016). Thioredoxins are ≈12 kDa disulfide oxidoreductases that utilize a pair of cysteines in the active site, Cys-X-X-Cys (CXXC), where X is any amino acid, to reduce disulfide bonds. As shown in **Figure 1B**, the N-terminal, more solvent exposed, cysteine is referred to as the “attacking” cysteine, which initiates disulfide reduction via disulfide exchange, forming a mixed disulfide intermediate between the target protein and the Trx. This is followed by attack of the C-terminal “resolving” cysteine on the N-terminal Trx Cys, with the release of the dithiol reduced target protein, and oxidized Trx. The oxidized Trx is then turned over (re-reduced) by thioredoxin reductase [TrxB in *Staphylococcus aureus* (*S. aureus*)] using a similar disulfide exchange mechanism with the reduced flavin adenine dinucleotide (FADH_2_) used to re-reduce TrxB disulfide and reduced nicotinamide adenine dinucleotide phosphate (NADPH) used as the final electron donor to reduce oxidized FAD (**Figure 1A**).

Mammalian Trx is capable of reducing a persulfide formed on the single reduced Cys of human serum albumin (HSA-SSH) (Wedmann et al., 2016) and the active site Cys of glyceraldehyde-3-phosphate dehydrogenase (GAPDH-SSH) (Ju et al., 2016). The thioredoxin-like protein TrxP in *S. aureus* is also capable of reducing the persulfide on lactate dehydrogenase-2 (Ldh2) (Peng et al., 2017b) *in vitro*. The active site cysteines of mammalian thioredoxin were reported to be important for the reduction of a protein persulfide, since mutation of the attacking cysteine of Trx1 weakens the binding affinity to persulfidated GAPDH (Ju et al., 2016). *In vivo*, inhibition of *trx* mRNA expression or Trx enzyme activity increases the extent of proteome persulfidation (Ju et al., 2016), suggesting that Trx system can serve to regulate endogenous proteome persulfide loads in mammalian cells. Since protein persulfides can be considered hydrodisulfides, an analogous Trx-like catalytic mechanism may well be operative, in the reduction of protein persulfides except that formation of the initial mixed disulfide intermediate gives rise to the release of H_2_S (**Figure 1B**). The ultimate cellular fate of this H_2_S is not known but may well be assimilated by low molecular weight (LMW) thiols via a number of enzymatic and non-enzymatic processes (Peng et al., 2017a; Shen et al., 2018) or used directly in Fe-S cofactor biogenesis.

*S. aureus* is a major nosocomial human pathogen which causes skin infections, respiratory infections and extensive mortality, with oxidation-reduction (redox) homeostasis an emerging antibacterial target against a growing resistance to commonly deployed antibiotics (Liu et al., 2016). We previously profiled proteome *S*-sulfuration in *S. aureus* and identified a variety of enzymes and transcriptional regulators that are subjected to *S*-sulfuration in cells. For example, *S*-sulfuration of an oxidation-sensing SarA family repressor, MgrA, reduces its DNA binding affinity and *S*-sulfuration of the active site of *S. aureus* GAPDH inhibits its activity (Peng et al., 2017b). We also identified two previously uncharacterized thioredoxin-like proteins that are *S*-sulfurated in *S. aureus* that we named TrxP and TrxQ, which are capable of reducing protein persulfides using the persulfidated rhodanese domain of the sulfurtransferase CstA, designated CstA^Rhod^-SSH (Higgins et al., 2015) as a model substrate (Peng et al., 2017b). The Δ*trxP*/Δt*rxQ* double mutant *S. aureus* strain exhibits a growth lag phenotype when exposed to exogenous sulfide stress, which implicates TrxP and/or TrxQ as candidate proteome depersulfidases in *S. aureus* (Peng et al., 2017b).

The relatively modest activity of TrxP and TrxQ toward CstA^Rhod^-SSH suggests that CstA^Rhod^-SSH, as a *bona fide* sulfurtransferase likely involved in persulfide shuttling (Higgins et al., 2015), may not be an authentic substrate for either enzyme. In fact, it seems possible that the individual thioredoxins in *S. aureus* may catalyze the persulfide reduction of distinct classes of target substrates given the economy of the bacterial genome. Therefore, to better understand the specific functions of TrxP and TrxQ, it is necessary to profile their substrates *in vivo*. Three analytical techniques have been used previously to identify candidate thioredoxin substrates. The first method is an indirect identification of thioredoxin substrates by differential labeling of thiols in different oxidation states to enrich and profile the oxidized cysteine residues in a wild-type vs. Trx-deficient cells (Leichert and Jakob, 2004; Le Moan et al., 2006; Hägglund et al., 2008). The second method relies on the purification of proteins bound to thioredoxin by tandem affinity purification, which suffers from the limitation that proteins that interact most strongly with thioredoxins are not necessarily Trx substrates (Kumar et al., 2004). The third method is mechanism-based, where the resolving cysteine of a thioredoxin is mutated to an alanine or serine, and an affinity-tagged, mutated thioredoxin is expressed *in vivo* and thus forms a stable intermolecular disulfide with the substrate as a mechanistic intermediate (see **Figure 1B**); this substrate is enriched by affinity capture and identified by LC-MS/MS (Motohashi et al., 2009; Nakao et al., 2015).

In this work, we employ the mechanism-based profiling method by constitutive expression of a specific FLAG-tagged Trx in a wild-type or Δ*trxP*/Δt*rxQ* mutant *S. aureus* strain to identify authentic cellular substrates of TrxA, TrxP, and TrxQ under conditions of exogenous sulfide stress (Luebke et al., 2014). We find that TrxA, TrxP, and TrxQ are characterized by distinct groups of candidate substrates, the majority of which were previously mapped as sites of proteome *S*-sulfuration (Peng et al., 2017b). Biochemical characterization of a representative enzyme target, pyruvate kinase, which forms crosslinks with TrxP in cells, provides new insights into target protein specificity in the cell. Pyruvate kinase is regarded as a major cellular interactome “hub” protein in methicillin-resistant *S. aureus*, and as key glycolytic, homo-oligomeric enzyme that is subject to extensive metabolic regulation, and is a novel drug target (Cherkasov et al., 2011; Zoraghi et al., 2011). Our studies suggest that reversible *S*-sulfuration of pyruvate kinase may well be regulatory in cells, as previously established for the human enzyme (Longen et al., 2016). The function of TrxQ, in contrast, remains mostly undefined, but may play a more specialized cellular role in Fe-S biogenesis.

## Materials and Methods

### Plasmid Construct and Bacterial Strains

C-terminally FLAG-tagged *trxP* was cloned into pOS1 (Luebke et al., 2014) using the *Nde*I and *Bam*HI restriction sites with primers 5′-CCCCCCCATATGCAATCAATCAAAAGTA-ATGAA-3′ and 5′-CCCCCCGATCCTTATTTATCATCATCATCTTTATAATCTTTAAAAGTTT-CTGCTAAAAA-3′. The resolving cysteine (C32) was mutated to serine (C32S) by site-directed mutagenesis using primers 5′-GAGGCAGGATGGAGCCCAGACTGTCGTG-3′ and 5′-CACGACAGTCTGGGCTCCATCC-TGCCTC-3′ to create pOS1_TrxP^CS^. Similarly, N-terminally FLAG-tagged *trxQ* was cloned into pOS1 using primers 5′-CCCCCCCATATGGATTATAAAGATGATGATGATAAAATGAATAATTCATTAGACATCAAA-3′ and 5′-CCCCCCGATCCC-TATTTTAAATTTTCTAACAAATCAGTC-3′. The resolving cysteine (C31) was mutated to serine (C31S) using primers 5′-CAACGTGTGGTACTTCTAAGGTTTCAGAAAG-3′ and 5′-CTTTCTGAAACCTTAGAAGTACCACACGTTG-3′ to create pOS1_TrxQ^CS^. In addition, C61 was mutated to serine (C61S) using primers 5′-CTTTTATCCTCAGTTTTCTAAAGATATGCAAATC-3′ and 5′-GATTTGCATATCTTTAGAAAACTGAGGATAAAAG-3′ to pOS1_TrxQ^CSS^. N-terminally FLAG-tagged *trxA* was cloned into pOS1 using primers 5′-CCCCCCCATATGGATTATAAAGATGATGATGATAAAGCAATCTAAAAGTAACAGATGC-3′ and 5′-CCCCCCGATCCTTATAAATGTTTATCTAAAACTTCAGCTAAG-3′. The resolving cysteine (C32) was mutated to serine (C32S) using primers 5′-CATGGTGTGGTCCATC-TAAAATGATCGCTC-3′ and 5′-GAGCGATCATTTTAGATGGACCACACCATG-3′ to create pOS1_TrxA^CS^.

These pOS1_Trx^i^ constructs were first transformed to *S. aureus* RN4220 by electroporation and colonies selected for chloramphenicol (Cm) resistance (10 μg/mL). Plasmids were extracted from Cm-resistant colonies by QIAprep spin miniprep kit following a 30 min digestion with lysostaphin at 37°C, and then transformed into the *S. aureus* Newman strain by electroporation (Shen et al., 2016). The integrity of plasmids derived from *S. aureus* Newman transformants that grew in presence of 10 μg/mL Cm were confirmed by sequencing and purified. pOS1_TrxP^CS^ and pOS1_TrxQ^CSS^ were transformed into the insertionally inactivated *trxP trxQ* double mutant strain (denoted here as Δ*trxP*/Δ*trxQ*) (Peng et al., 2017b), while the pOS1_TrxA^CS^ plasmid was transformed into wild-type *S. aureus* Newman strain.

### Mechanism-Based Thioredoxin Substrate Profiling by MS

pOS1_Trx^i^-transformed *S. aureus Newman* strains were grown in 10 mL TSB medium overnight. Cells were then pelleted and resuspended in a chemically defined minimal growth medium (HHWm) (Luebke et al., 2014) supplemented with 0.5 mM thiosulfate and 0.5 mM methionine to initiate growth of the culture at OD_600_ = 0.007. All cultures were grown in loosely capped 50 mL Falcon tubes at 37°C with shaking at 200 rpm. Na_2_S was added to these cultures at a final concentration of 0.2 mM when the OD_600_ reached 0.2 as done previously for previously described *S*-sulfuration profiling experiments (Peng et al., 2017a,b). Cultures of 140 mL were collected 20 min after the addition Na_2_S and centrifuged at 6000 rpm for 20 min. Supernatants were discarded and the cell pellets washed with PBS and pelleted twice.

Cell pellets were resuspended in 1 mL lysis buffer (50 mM Tris-HCl, 150 mM NaCl, 1 mM EDTA, pH 7.4) and processed by five freeze-thaw cycles using liquid nitrogen and a 37°C water bath. The resuspended pellet was transferred to a 2 mL lysing matrix B (MP Biomedicals) tube and shaken at 6.8 m/s for 60 s, 4°C, then spun at 13,000 rpm for 15 min, 4°C. The supernatant of ≈500 μL was added to 40 μL Anti-FLAG M2 affinity gel (Sigma-Aldrich), which was prepared by pre-washing three times with 0.5 mL TBS buffer (50 mM Tris-HCl, 150 mM NaCl, pH 7.4) in a 1.5 mL microcentrifuge tube at 4°C. The cell lysate was incubated with the anti-FLAG resin for 2 h on a roller shaker at 4°C, the tube centrifuged at 5000 *g* for 30 s, and the supernatant collected as the flow-through (FT) sample. The resin was then washed three times with 0.5 mL wash buffer (50 mM Tris-HCl, 150 mM NaCl, 1 mM EDTA, 1% Triton X-100, pH 7.4), and once with 0.5 mL lysis buffer at 4°C. The FLAG-tagged Trx-substrate protein conjugates were eluted by addition of 103 μL 5 mg/mL 3x-FLAG peptide in lysis buffer for 30 min on a roller shaker at 4°C, followed by centrifugation. 10 μL of total cell lysate, FT and wash samples, and 2 μL of eluted fraction were run on an SDS-PAGE gel to evaluate the expression of the FLAG-tagged Trx and the progress of the FLAG-enrichment strategy was confirmed by Western blot using anti-FLAG antibody (described below). The remainder of the sample was digested by trypsin in presence of 2 M urea in the same buffer overnight, with the digestion quenched by addition of 1 μL 10% trifluoroacetic acid (TFA). Peptide samples were desalted by C18 ziptip using a standard protocol, dried and resuspended in 0.1% formic acid for LC-MS/MS analysis performed in the Laboratory for Biological Mass Spectrometry in Indiana University (see below). Three biological replicates were conducted for each Trx substrate profiling experiment.

### Detection of FLAG-Tagged Trx Proteins by Western Blot

Samples electrophoresed on an SDS-PAGE gel were transferred to a polyvinylidene difluoride (PVDF) membrane pre-blocked with 1% serum in PBS-T (PBS with 0.1% Tween 20, pH 7.4), and subsequently probed with monoclonal anti-FLAG M2-peroxidase (HRP) antibody produced in mouse (Sigma) in PBS-T. Western blots were imaged by SuperSignal^TM^ West Pico Chemiluminescent Substrate (Thermo Scientific) and exposed to film.

### UPLC-ESI-MS/MS Detection of Crosslink Peptides

Peptide digests were analyzed using an Orbitrap Fusion Lumos mass spectrometer (Thermo Scientific, Bremen, Germany) equipped with an Easy-nLC 100 HPLC system. Briefly, 1 μg peptide was loaded onto Acclaim PepMap^TM^ 100 C18 trap column (75 μm × 20 mm, 3 μm, 100 Å) for 6 min in 0.1% formic acid and further chromatographed on an Acclaim PepMap^TM^ RSLC C18 analytical column (75 μm × 150 mm, 2 μm, 100 Å) using an acetonitrile-based gradient (Solvent A: 0% acetonitrile, 0.1% formic acid; Solvent B: 80% acetonitrile, 0.1% formic acid). The elution protocol was as follows: 0–1 min, 3–7% B, linear gradient; 1–19 min, 7–40% B, linear gradient; 19–21 min, 40–100% B, linear gradient; 21–21.5 min, 100% B, linear gradient; 21.5–25 min, 100–3% B, isocratic followed by re-equilibration to 3% B. Data were collected by the Xcalibur System and converted into Mascot Generic Format by Proteome Discoverer 2.0. The extracted peak list was analyzed using Protein Prospector (UCSF).

### Protein Purification

The *S. aureus* strain Newman Trx coding sequences were subcloned into a pHis-parallel vector (Sheffield et al., 1999) to create pHis_TrxA, pHis_TrxP, and pHis_TrxQ, and subjected to site-directed mutagenesis using the primers described above to create pHis_TrxA^CS^, pHis_TrxP^CS^, pHis_TrxQ^CS^, and pHis_TrxQ^CSS^. The purification of His-tagged TrxA^CS^, TrxP^CS^, TrxQ^CS^, and TrxQ^CSS^ was carried out essentially as described previously for their wild-type counterparts (Peng et al., 2017b). In addition, the coding sequences of genes encoding a number of candidate Trx target substrates, including pyruvate kinase (*pykA* or PK) (Zoraghi et al., 2011), 3-hydroxy-3-methylglutaryl coenzyme A synthase (*mvaS*) (Theisen et al., 2004) and uracil phosphoribosyltransferase (*upp*) were subcloned into the pHis-parallel vector between the *Nco*I and *Hind*III restriction sites and transformed into *Escherichia coli* BL21 DE3 *E. coli* cells. These cultures were grown in LB at 37°C, induced with 0.5 mM IPTG at an OD_600_ of 0.7–0.9, expressed at 16°C overnight, and cell pellets collected by low-speed centrifugation. All candidate Trx target proteins were purified using a similar protocol that involved initial solubilization of the pellet in degassed buffer A (25 mM Tris, pH 8.0, 500 mM NaCl, 20 mM imidazole, 2 mM TCEP), followed by sonication. The cell lysate was centrifuged and the supernatant subjected to nucleic acid precipitation with the addition of 0.15% (w/v) polyethyleneimine (PEI), followed by centrifugation at 7,000 rpm for 15 min, 4°C. Proteins were precipitated from the supernatant with the additional of 70% (w/v) ammonium sulfate for 1 h, 4°C, and the pellets resuspended in buffer A. The resuspended ammonium sulfate pellets were filtered through a 0.2 μm filter and loaded onto a pre-equilibrated Ni-NTA column in buffer A. The column was washed with buffer A containing 50 mM imidazole, followed by elution with a gradient of 0–80% buffer B (25 mM Tris, pH 8.0, 500 mM NaCl, 500 mM imidazole, 2 mM TCEP). Recombinant proteins were further purified by gel filtration chromatography on a HiLoad^TM^ 16/600 Superdex^TM^ column. Fractions of >90% purity (estimated by inspection of SDS-PAGE gels) were pooled and stored at -80°C with 15% glycerol until use. The integrity of all candidate Trx substrate proteins was confirmed by matrix-assisted laser desorption ionization (MALDI) mass spectrometry, with reduced thiol contents confirmed by DTNB analysis.

### Generation of Protein Persulfides and Detection of the Site(s) of *S*-Sulfuration

Recombinant proteins were buffer exchanged into degassed 25 mM Tris-HCl, pH 8.0, 200 mM NaCl in the glove box. A 200-fold molar excess of Na_2_S_4_ was added to 10–50 μM protein, incubated overnight at ambient temperature, and excess Na_2_S_4_ removed by buffer exchange against 25 mM Tris-HCl, pH 8.0, 200 mM NaCl, 5 mM EDTA in the glove box. The site(s) of *S*-sulfuration was confirmed using the LC-MS/MS workflow described previously (Peng et al., 2017b). Briefly, 20 μM of 25 μL *S*-sulfurated protein was diluted with 75 μL of 8 M urea in 100 mM ammonium bicarbonate buffer and alkylated with 20-fold molar excess of biotinylated iodoacetamide (b-IAM) for 90 min anaerobically in the dark at ambient temperature. Proteins were then digested with trypsin at 37°C overnight following the addition of 200 μL 100 mM ammonium bicarbonate buffer to the samples. These peptide samples were desalted by a C18 Omix column following a standard protocol and dried in Speedvac^TM^ concentrator. Cysteine-containing peptides labeled by b-IAM were then enriched on a Neutravidin resin in 100 mM ammonium bicarbonate. Non-specifically adsorbed peptides were washed from the column with 2 M NaCl in 100 mM ammonium bicarbonate, followed by elution with 10 mM TCEP at 37°C, alkylation with iodoacetamide (IAM), desalting with a C18 ziptip, and analysis by LC-MS/MS as describe above.

### Thioredoxin Activity Assays

Recombinant His-tagged TrxA, TrxP, TrxQ, C61S TrxQ, and TrxB, purified as described earlier (Peng et al., 2017b), were buffer exchanged into the reaction buffer (25 mM Tris-HCl, pH 8.0, 200 mM NaCl, 5 mM EDTA) in the glove box. 100 μL of an enzyme solution containing 2 μM TrxB and 10 μM of a Trx in reaction buffer was mixed with 100 μL of a persulfidated substrate solution containing 800 μM NADPH and variable concentrations of the substrate protein in a 96-well plate out of the glove box. The absorbance at 340 nm was monitored using a Synergy Neo2 plate reader (BioTek) at ambient temperature every 5 s over a 3 min time period. The concentration of NADPH was calculated using a standard curve measured by the plate reader, with initial velocity (in μmol s^-1^ mg^-1^) obtained by fitting the data to a linear function. The kinetic data shown here for CstA^Rhod^-SSH as a substrate was previously reported (Peng et al., 2017b) and shown here only to facilitate comparison to PykA-SSH.

### TrxA^CS^ and TrxP^CS^ Substrate Protein Crosslinking *in vitro* and TrxQ Persulfidation Experiments

To validate the results the *in vivo* thioredoxin profiling experiments, 10 μM TrxA^CS^ or TrxP^CS^ was anaerobically incubated with 10 μM persulfidated PykA (PykA-SSH) in 50 μL total volume for 1 h at ambient temperature, and the samples analyzed as described above. For TrxQ persulfidation experiments, 20 μM TrxQ was anaerobically incubated with a 20-fold molar excess of Na_2_S_4_ in 50 μL for 1 h at ambient temperature, with the reaction capped with IAM, digested with trypsin overnight at 37°C and quenched by 1% TFA. Peptides were desalted by C18 ziptip using a standard protocol, with the site(s) of persulfidation detected by mass spectrometry as previously described (Shen et al., 2016).

## Results and Discussion

### Multiple Thioredoxin-Like Proteins in Bacteria

**FIGURE 2:**
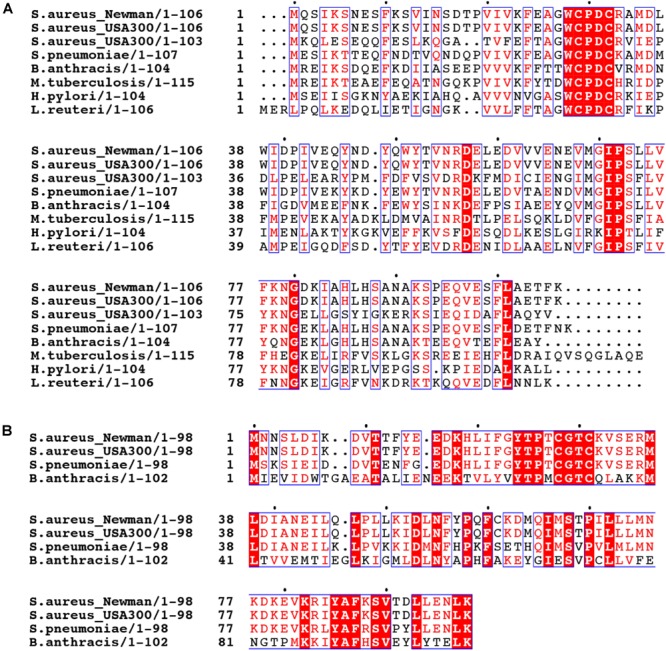
Multiple sequence alignment of thioredoxin-like proteins in selected bacteria. The sequences of *S. aureus* strain Newman TrxP harboring a (W**C**PD**C**) active site **(A)** and TrxQ (**C**GT**C**) **(B)** are aligned with Trx-like proteins encoded by other *S. aureus* strains, including two *S. aureus* USA300 strains, and other bacteria including *Streptococcus pneumoniae* D39 (*S. pneumoniae*), *Bacillus anthracis* (*B. anthracis*), *Mycobacterium tuberculosis* (*M. tuberculosis*), *Helicobacter pylori* (*H. pylori*), and *Lactobacillus reuteri* (*L. reuteri*).

Trx family proteins include enzymes that catalyze protein thiol-disulfide exchange reactions in substrate proteins and harbor a CXXC motif in the active site. The canonical thioredoxin possesses a W**C**GP**C** active site around residue 30 (**Figure 2** and **Supplementary Figure S1**). The *S. aureus* strain *Newman* encodes multiple Trx-like proteins in addition to a canonical thioredoxin, TrxA, including TrxP, TrxQ and NWMN_1637 (Peng et al., 2017a). Each is ≈100 residues in length but harbors a **C**XX**C** motif that differs from the canonical W**C**GP**C** site in TrxA (Peng et al., 2017b). Whether NWMN_1637 has thioredoxin activity is unknown. All *Sau* Trx-like proteins are expected to adopt a canonical Trx fold characterized by a core of five β-sheets surrounded by four α-helices, and this is known for both *Sau* TrxA (Roos et al., 2007) and TrxP (Peng et al., 2017b). It seems possible that individual Trx-like proteins have functions distinct from that of TrxA, or possess different substrate selectivities.

The presence of multiple Trx-like proteins appears to be a common feature of other *S. aureus* strains or other bacterial pathogens. When the TrxP and TrxQ are used as query in a BLAST search against the genomes of a selected bacteria, a canonical TrxA as well as TrxP-like (W**C**PD**C**) and TrxQ-like (**C**GT**C**) proteins are often found (**Figure 2**). We note that TrxQ harbors an active site sequence that when transplanted into *Sau* TrxA lowers the driving force for substrate reduction (Roos et al., 2007). In any case, a number of *Firmicutes*, including *Bacillus anthracis* and *Lactobacillus reuteri*, harbor TrxP-like proteins, as does a representative of Gram-negative bacterium, *Helicobacter pylori* (**Figure 2**). However, other Trx-like proteins with distinct active site signatures are also found (**Supplementary Figure S1**). The methicillin-resistant *S. aureus* (MRSA) strain FPR3757 and *B. anthracis* only encode TrxP- and/or TrxQ-like proteins in addition to the canonical thioredoxin (**Supplementary Figure S1**). However, other bacterial genomes investigated here, including *Enterococcus faecalis, Streptococcus pneumoniae, Pseudomonas aeruginosa*, and *Mycobacterium tuberculosis*, encode thioredoxin-like proteins which harbor distinct active sites, including CGDC, CSVC, CGHC, and CAPC, with some encoding TrxP- or TrxQ-like proteins, with most of unknown function (**Supplementary Figure S1**).

### Mechanism-Based Substrate Profiling of TrxA, TrxP, and TrxQ in *S. aureus*

**FIGURE 3:**
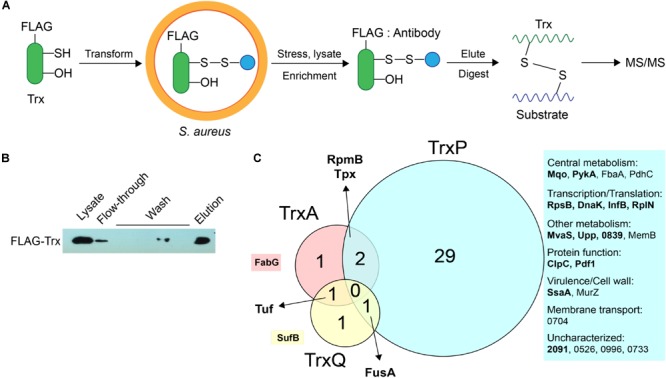
Mechanism-based Trx substrate profiling experiment in *S. aureus*. **(A)** Flowchart of the profiling experiment. **(B)** SDS-PAGE derived progress of the profiling experiment monitored by Western blotting using an anti-FLAG antibody. **(C)** Venn diagram showing different groups of cysteine residues in proteins are identified as candidate TrxA, TrxP, and TrxQ substrates, respectively. Proteins observed in cells as *S*-sulfurated in previous work (Peng et al., 2017b) are highlighted in bold. All candidate substrates identified as crosslinked peptides are labeled next to the Venn diagram, with additional information provided in **Table 1**. The 4-digit locus tag numbers are used to represent the proteins that do not have a functional or designated name (“NWMN_” omitted).

We previously showed that TrxA, TrxP, and TrxQ are capable of reducing both a protein persulfide and a protein disulfide bond using the multidomain sulfurtransferase, CstA, as a model substrate (Peng et al., 2017b). However, TrxA exhibited a higher specific activity toward the disulfide relative to TrxP and TrxQ, while all three enzymes showed comparable activities toward that single persulfide substrate (Peng et al., 2017b). Since the Δ*trxP*/Δ*trxQ* strain shows a growth phenotype upon exposure to exogenous sulfide stress, and TrxP and TrxQ are detected as *S*-sulfurated in cells under the same conditions while TrxA is not, we proposed that TrxP and TrxQ are specifically involved in cellular protein persulfide reduction while TrxA catalyzes disulfide reduction (Peng et al., 2017b). To obtain support for this idea, while also exploring potential substrate specificity of these three Trx in cells, we sought to profile candidate Trx substrates under conditions of sulfide stress *in vivo*. In this mechanism-based profiling method (**Figure 3A**), candidate Trx substrates are trapped by forming an intermolecular disulfide bond with the attacking cysteine of a specific FLAG-tagged Trx, which does not resolve since the resolving cysteine is mutated to a serine. Since TrxQ has an additional single cysteine beyond the active site (C61), this cysteine was also mutated to a serine to avoid potential crosslinks to this Cys. C61S TrxQ has steady state kinetic parameters in persulfide reduction that appear comparable to that of wild-type TrxQ (**Supplementary Figure S2**). Cell lysate samples were collected from mid-log cultures following addition of 0.2 mM Na_2_S as done previously for the proteome *S*-sulfuration profiling experiments (Peng et al., 2017b). FLAG-tagged Trx-substrate conjugates were enriched on an anti-FLAG affinity resin, eluted and digested into peptides by trypsin. Only those substrate peptides that formed disulfide bonds with a Trx and are identified with high confidence by high resolution LC-MS/MS (±5 ppm mass accuracy) are reported here.

The utility of this experimental strategy was first confirmed by anti-FLAG Western blotting and SDS-PAGE analysis (**Figure 3B**). These results show that FLAG-tagged Trx is constitutively expressed and detected in the cell lysate, and that FLAG-tagged Trx remains bound to the resin during wash step, and is subsequently eluted via addition of FLAG peptide (**Figure 3B**). Eluted fractions from three biological replicate lysates were then subjected to bottom-up, high-resolution LC-MS/MS and taken as representative of cellular substrate profiles for each Trx. All three Trx are constitutively expressed from the same plasmid, and are therefore expected to be expressed at similar concentrations (see lane 1, **Figure 3B**). To approximate the relative amounts of FLAG-tagged TrxA, TrxP, and TrxQ pulled down in each of experiment, the total MS signal intensity corresponding to all identified peptides from each Trx protein was calculated from all three replicate experiments (**Supplementary Figure S3**). These data reveal that TrxP was pulled-down to an extent that is lower than the other Trx proteins, approximately one-half that of TrxQ, and ≈8-fold less than TrxA. Thus, the efficiency of detecting TrxP crosslinks is in fact somewhat lower than our ability to detect TrxQ and TrxA crosslinks.

TrxA substrate profiles obtained from a wild-type *S. aureus* Newman strain background (containing endogenous TrxA) identified four substrates in total, all of which were previously identified as *S*-sulfurated in cells (Peng et al., 2017b) (highlighted in bold in **Figure 3C**). TrxQ substrate profiles derived from a Δ*trxP*/Δ*trxQ S. aureus* Newman strain background identified just three candidate substrates all of which were detected as *S*-sulfurated in cells, including two abundant translation factors (elongation factor-G, EF-Tu) shared in common with the TrxP and TrxA profiles, respectively. In contrast, TrxP substrate profiles derived from the same Δ*trxP*/Δ*trxQ S. aureus* Newman strain background revealed crosslinks to 32 different cysteines on 25 different proteins, with 29 of these cysteines unique to TrxP and 69% previously detected as *S*-sulfurated in cells (**Table 1** and **Figure 3C**). These 25 candidate TrxP substrate proteins include enzymes involved in central metabolism including the major glycolytic enzymes pyruvate kinase (PykA), fructose-1,6-bisphosphate aldolase (Ziveri et al., 2017), and the pyruvate dehydrogenase complex, proteins and enzymes involved in transcription and translation, virulence factors (SsaA), cell wall synthesis (MurZ) and cytoplasmic proteins associated with putative membrane transporters (**Figure 3C**). Thus, in contrast to TrxQ and TrxA, TrxP depersulfidase activity appears to possess a significant metabolic and regulatory footprint under conditions of sulfide and RSS stress. Although it is also formally possible that cysteine-containing peptides of TrxP and a crosslink with TrxP are intrinsically easier to detect or sequence by tandem MS than those peptides derived from TrxQ or TrxA, this seems unlikely given our quantitation of peptides eluted from the anti-FLAG column, in which TrxP-derived peptides were least abundant (**Supplementary Figure S3**).

**Table 1:** Sites of intermolecular disulfide crosslinking from the Trx profiling experiments^a^.

Locus tag	Gene name	Protein	Cys residue #	*S*-sulfurated in cells?	#^b^	Conserved in Firmicutes^c^	Active site solvated or buried	Structure^d^
**TrxA**								
**NWMN_0510**	***tuf***	**Elongation factor Tu**	**82**	**Y**	**1**	**Y**	**Solvated**	**1EFC *Eco***
**NWMN_1607**	***tpx***	**Probable thiol peroxidase**	**60**	**Y**	**2**	**Y**	**Active site**	**3P7X *Sau***
NWMN_0071	*fabG*	Glucose/ribitol dehydrogenase	155	Y	1	Y/N	Buried	1GEG
**NWMN_1134**	***rpmB***	**50S ribosomal protein L28**	**5**	**Y**	**1**	**Y**	**ND^e^**	**4WCE *Sau***
**TrxP**								
NWMN_0487	*clpC*	ATP-dependent Clp protease ATP-binding subunit	311	Y	1	Y	Buried	3J3S *Bsu*
NWMN_1592	*pykA*	Pyruvate kinase	8, 144, 410	Y	4	Y (8) Y/N (144,410)	Solvated (410)	3T05 *Sau*
NWMN_2446	*mvaS*	3-hydroxy-3-methylglutaryl coenzyme A synthase	111	Y	3	Y	Active site	1XPK *Sau*
NWMN_2448	*clpC*	ATP-dependent Clp protease ATP-binding subunit	295	Y	2	Y/N	ND^e^	3J3S *Bsu*
NWMN_2091	*–*	Uncharacterized	242	Y	2	Y	Solvated	4HFJ
NWMN_2504	*mqo*	Probable malate:quinone oxidoreductase	62§, 275, 373§	Y (62,275)	3	Y (62,275)	–	–
**NWMN_0509**	***fusA***	**Elongation factor G**	**258, 390, 473, 596**	**Y (258,390,473)**	**5**	**Y(596) Y/N others**	**Buried (258, 390)**	**2XEX *Sau***
**NWMN_1134**	***rpmB***	**50S ribosomal protein L28**	**5**	**Y**	**1**	**Y**	**ND^e^**	4WCE *Sau*
NWMN_1166	*rpsB*	30S ribosomal protein S2	191	Y	1	Y	Solvated	5NJT *Bsu*
NWMN_1483	*dnaK*	Chaperone protein DnaK	15	Y	1	Y	Solvated	2V7Y
NWMN_2016	*upp*	Uracil phosphoribosyltransferase	159	Y	1	Y	Buried	1I5E
NWMN_2199	*ssaA*	Secretory antigen SsaA	171§	Y	1	Y	Solvated	2K3A
NWMN_1178	*infB*	Translation initiation factor IF-2	633§	Y	2	Y	Buried	5ME0
NWMN_2142	*rplN*	50S ribosomal protein L14	41	Y	2	Y/N	Solvated	4WCE *Sau*
NWMN_0839	*–*	Fumarylacetoacetate hydrolase family	200§	Y	1	Y/N	–	–
NWMN_0957	*pdf1*	Peptide deformylase	111§	Y	1	Y	Active site	1LQW *Sau*
**NWMN_1607**	***tpx***	**Probable thiol peroxidase**	**60§**	**Y**	**2**	**Y**	**Active site**	**3P7X *Sau***
NWMN_0704	*–*	ABC transporter ATP-binding protein	48	N	3	Y/N	–	–
NWMN_0915	*memB*	1,4-dihydroxy-2-naphthoyl-CoA synthase	129	N	4	Y	Buried	2UZF *Sau*
NWMN_2029	*fbaA*	Fructose-bisphosphate aldolase	93§	N	2	Y	Solvated	4TO8 *Sau*
NWMN_0526	*–*	Uncharacterized (glycosyl transferase family 1)	282§	N	5	N	–	–
NWMN_0996	*–*	Uncharacterized	95§	N	1	Y/N	–	–
NWMN_0961	*pdhC*	Dihydrolipoamide acetyltransferase component of pyruvate dehydrogenase complex	346§	N	1	Y	ND^e^	3DUF
NWMN_2028	*murZ*	UDP-*N*-acetylglucosamine 1-carboxyvinyltransferase 2	376§	N	5	Y/N	Buried	3ZH3 *Spn*
NWMN_0733	*–*	Nucleotide-binding protein	262§	N	3	Y	Solvated	5O5Q *Eco*
**TrxQ**								
**NWMN_0509**	***fusA***	**Elongation factor G**	**258§**	**Y**	**5**	**Y/N**	**Buried**	**2XEX *Sau***
**NWMN_0510**	***tuf***	**Elongation factor Tu**	**82**	**Y**	**1**	**Y**	**Solvated**	**1EFC *Eco***
NWMN_0789	*sufB*	Fe-S assembly protein SufB	302	Y	3	Y/N	Buried	5AWF *Eco*

*^*a*^Those entries in bold were observed in multiple Trx profiling experiments carried out with different FLAG-tagged Trx. All crosslinks reported here were detected in at least two of the three biological replicates unless otherwise indicated by a §symbol beside the Cys residue number. ^*b*^Number of total cysteines in this protein; ^*c*^Y, invariant in Firmicutes; Y/N, conserved in Firmicutes. ^*d*^Bacterial origin of the structure shown only if derived from S. aureus (Sau), B. subtilis (Bsu) S. pneumoniae (Spn), or E. coli (Eco); all others not indicated. ^*e*^ND, indicated cysteine not found in the structure*.

These profiling data reveal that TrxA, TrxP, and TrxQ share no common candidate substrates with only a few common substrates for any one pair of thioredoxins. However, the majority of these common substrates are cell-abundant proteins, including EF-G, EF-Tu, the ribosomal protein RpmB (L28) and the thiol-dependent peroxidase or peroxiredoxin (Tpx) (**Figure 3C**). Thioredoxins are well known to provide electrons to Tpx to reduce reactive oxygen and reactive nitrogen adducts (Lu and Holmgren, 2014), and this and previous work (Peng et al., 2017b) suggests the same is true of persulfide adducts. This reaction, however, may not be Trx-specific. In addition, there is not a 1:1 correlation between those proteins detected as Trx substrates here vs. those previously identified as *S*-sulfurated in cells (Peng et al., 2017b) (**Table 1**). These proteins (1) may be present in cells but below the detection limit of the global profiling method used previously; (2) may be cell-abundant proteins that crosslink with Trx non-specifically, e.g., ribosomal proteins L28 and L14; or (3) harbor modifications other than *S*-sulfuration, e.g., *S-*thiolations, oxidative (RSOH) or nitrosative (RSNO) modifications, that may also be reduced by thioredoxins (Loi et al., 2015). The profiling experiments described in this work cannot distinguish among these possibilities.

Further consideration of these Trx-conjugates reveals that 60% of the identified cysteines are invariant in Firmicutes, with some in the active site of the enzyme (**Table 1**). For example, C111 of 3-hydroxy-3-methylglutaryl coenzyme A synthase (MvaS), which catalyzes the rate-limiting step of cholesterol synthesis (Theisen et al., 2004), is reproducibly detected as a candidate substrate for TrxP (**Table 1**), and represents the active site cysteine. However, it proved difficult to persulfidate MvaS *in vitro* with polysulfide or coenzyme A persulfide, thus preventing us from validating the TrxP-dependent persulfide reduction of MvaS (data not shown). An interesting example of substrate specificity is the metabolic enzyme, pyruvate kinase (PykA), which has four cysteine residues (C8, C144, C266, and C410). Only C266 was not detected in the proteome *S*-sulfuration profiling experiments likely due to its short tryptic peptide (Peng et al., 2017b); in our Trx-substrate profiling experiment reported here, C8, C144, and C410 are identified as substrate sites for TrxP (**Table 1** and **Supplementary Figure S4**).

The unique TrxQ substrates perhaps suggest a more specialized role in the regulation of cellular metabolism relative to TrxP, including a potential role in iron-sulfur (Fe-S) biogenesis (SufB). SufB likely functions as part of a Suf BC_2_D scaffold that mediates SufC-catalyzed ATP hydrolysis-dependent gating of Fe-S cofactor assembly (Hirabayashi et al., 2015) (**Supplementary Figure S5**); however, recombinant *Sau* SufB when expressed in *E. coli* was insoluble (Dos Santos, 2017) and thus was not studied further.

### Catalytic Activities of TrxA, TrxP, and TrxQ Toward a Model Protein Persulfide Substrate

We chose tetrameric *S. aureus* PykA as a model substrate of known structure (Zoraghi et al., 2011) (**Supplementary Figure S4**) to investigate the specific activities of TrxA, TrxP, and TrxQ toward PykA protein persulfide reduction. PykA serves as an excellent model substrate since PykA was detected reproducibly only in replicate TrxP profiling experiments and three of the four Cys are sites of protein *S*-sulfuration *in vivo* (**Table 1** and **Supplementary Figures S4A,B**). Furthermore, mammalian PykA is readily persulfidated *in vitro* via reaction with inorganic polysulfides (Longen et al., 2016). The *S. aureus* PykA-SSH, persulfidated form of PykA, was generated *in vitro* and confirmed by our previously published enrichment procedure used for protein *S*-sulfuration profiling (Peng et al., 2017b). As expected, C8, C144, and C410 were detected as persulfidated, validating *S*-sulfuration on PykA in cells (**Supplementary Figure S6**). It is unknown if C266 is *S*-sulfurated using this assay since the peptide is too short to be detected in either the previous or this workflow (Peng et al., 2017b).

**FIGURE 4:**
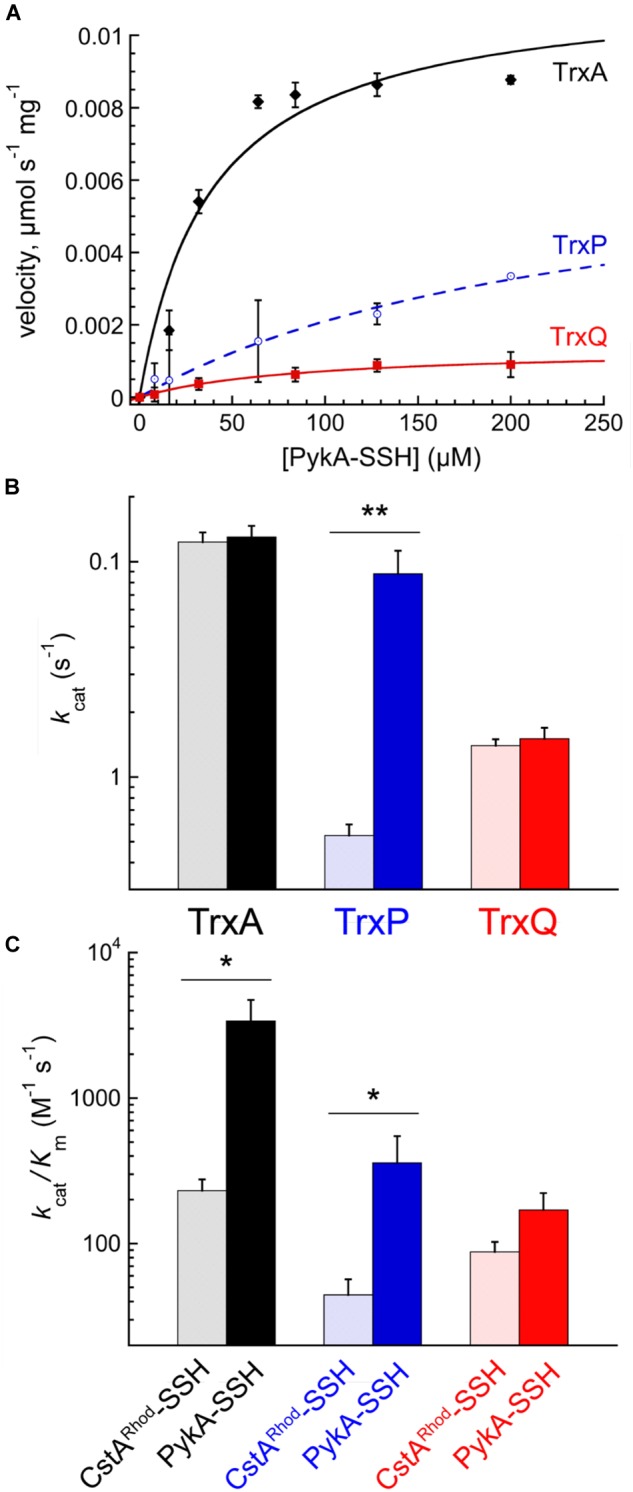
Reduction of PykA-SSH as a model substrate by TrxA, TrxP, and TrxQ. **(A)** Steady-state kinetics of the reduction of the of PykA-SSH plotted as a function of PykA-SSH substrate concentration for TrxA (*black* symbols), TrxP (*blue*) and TrxQ (*red*), with the continuous line a fit to the Michaelis–Menten model in each case and parameters summarized in **Table 2**. Triplicate experiments were conducted in each case, with the standard deviation of the mean of each data point shown by the error bars. **(B)** The apparent turnover numbers (*k*_cat_) of TrxA (*black triangles*), TrxP (*blue circles*) and TrxQ (*red squares*) acquired at 1 μM TrxB with PykA-SSH as a substrate (*filled* symbols) compared to CstA^Rhod^-SSH (*open* symbols) as a substrate taken from a previous report (Peng et al., 2017b). **(C)** The catalytic efficiencies, *k*_cat_/*K*_m_, of TrxA, TrxP, and TrxQ measured at 1 μM TrxB toward PykA-SSH as a substrate (*filled* symbols) compared to CstA^Rhod^-SSH as substrate (Peng et al., 2017b). ^∗^*p* ≤ 0.05, ^∗∗^*p* ≤ 0.01.

PykA-SSH prepared in this way was then used as a substrate in Trx-mediated persulfide reduction reactions, with the TrxA-, TrxP-, and TrxQ-catalyzed initial velocities obtained as a function of [PykA-SSH] at 1 μM thioredoxin reductase (TrxB) and saturating NADPH, and fit to the Michaelis–Menten model (**Figure 4A**) with parameters compiled in **Table 2**. TrxA, TrxP, and TrxQ all possess measurable activities toward PykA-SSH, with the rank order of the three Trx in terms of maximal velocity (*V*_max_) found to be TrxA > TrxP > TrxQ (**Figure 4A** and **Table 2**). We next compared these catalytic parameters with those obtained previously with another *S*-sulfurated substrate CstA^Rhod^-SSH (Peng et al., 2017b), which is not detected in any Trx profiling experiment (see **Table 1**). The apparent turnover number, *k*_cat_, increases ≈15-fold for TrxP only (**Figure 4B**). TrxQ, on the other hand, has a relatively low apparent turnover number (*k*_cat_) toward both CstA^Rhod^-SSH and PykA-SSH, neither of which are *bona fide* cellular substrates (**Figure 4B** and **Table 1**). These findings are consistent with the fact that PykA is an authentic *in vivo* substrate for TrxP while TrxQ is not (**Figure 3**), and predicated on the expectation that the activity would be higher with an authentic cellular substrate. Finally, the catalytic efficiencies (*k*_cat_/*K*_m_) of both TrxA and TrxP are also found to increase significantly when comparing PykA-SSH to CstA^Rhod^-SSH (**Figure 4C** and **Table 2**), traced to a lower *K*_m_ of TrxA for CstA^Rhod^-SSH measured previously (Peng et al., 2017b). These values of *k*_cat_/*K*_m_ for persulfide reduction are ≈100-fold lower that previously found for *E. coli* thioredoxin in disulfide reduction of a model substrate, insulin (Dyson et al., 1997), but for *S. aureus* TrxA, much of this difference is traced to a ≈40-fold higher *k*_cat_/*K*_m_ for a disulfide (*k*_cat_/*K*_m_ ≈ 10^5^ M^-1^ s^-1^) relative to a persulfide substrate (Peng et al., 2017b).

**Table 2:** Steady-state kinetic parameters of the TrxA-, TrxP-, and TrxQ-mediated persulfide reduction of PykA-SSH.

Enzyme	*K*_m_ (μM)	*V*_max_ (μmol s^-1^ mg^-1^)	*k*_cat_ (s^-1^)^a^	*k*_cat_/*K*_m_ (M^-1^ s^-1^)^a^
TrxA	39 ± 15	0.0114 ± 0.0014	0.130 ± 0.016	3400 ± 1400
TrxP	240 ± 110	0.0072 ± 0.0020	0.088 ± 0.024	360 ± 190
TrxQ	80 ± 25	0.0014 ± 0.0002	0.015 ± 0.002	170 ± 53

*^*a*^Kinetic parameters expressed relative to the concentration of the individual thioredoxin (5 μM), as indicated, at a fixed concentration of TrxB (1 μM), and 400 μM NADPH with [PykA-SSH] allowed to vary from 1 to 200 μM.*

In a further effort to recapitulate the cellular Trx substrate specificity in PykA *in vitro*, we purified non-resolving mutants of TrxA and TrxP, designated TrxA^CS^ and TrxP^CS^, respectively, and incubated each with PykA-SSH anaerobically, and analyzed these reaction mixtures by LC-MS/MS as described for the cell lysate experiments (**Figure 3**). Contrary to the *in vivo* substrate profiling results, both TrxA^CS^ and TrxP^CS^ showed crosslinks to the same three cysteine residues of PykA *in vitro* (**Supplementary Figure S6**), suggesting that the reduced complexity environment with the purified enzyme in isolation may not be a good model to enforce intrinsic substrate selectivity or TrxP vs. TrxQ. On the other hand, the intermolecular disulfide bond formed between the attacking cysteine on each Trx and PykA in the *in vitro* reaction confirms this conjugate as a mechanistic intermediate during persulfide reduction, exactly analogous to the initial steps of disulfide reduction by a canonical thioredoxin (**Figure 1B**).

**FIGURE 5:**
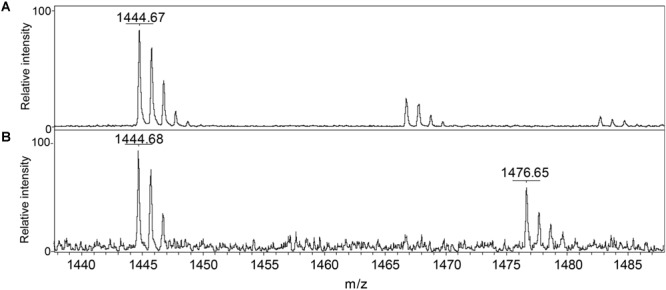
Detection of reduced and *S*-sulfurated TrxQ C61-containing peptide 52–62 using MALDI-MS. The theoretical *m/z* of reduced peptide is 1444.7 and sulfureted peptide is 1476.7. **(A)** Untreated TrxQ sample; **(B)** TrxQ incubated with a 20-fold molar excess of Na_2_S_4_ and analyzed (see **Supplementary Table S2**).

We conclude that *S. aureus* TrxA may be an all-purpose thioredoxin that is active against a wide range of oxidative modifications of proteome cysteines consistent with the known properties of the mammalian Trx (Lu and Holmgren, 2014). TrxP, on the other hand, is the major depersulfidase in *S. aureus* cells, particularly under conditions of the sulfide stress or RSS misregulation. The functional role of TrxQ in cells remains enigmatic, given the small number of candidate TrxQ substrates detected here and poor depersulfidase activities toward two non-cognate substrates (**Figure 3**). It also possible that TrxQ has maximal activity with another, as yet uncharacterized, thioredoxin reductase. However, it is interesting to note that it is C61, not the active site Cys pair in TrxQ, that is detected as *S*-sulfurated in cells (Peng et al., 2017b). *In vitro* experiments confirm this, since incubation of TrxQ with Na_2_S_4_ reveals significant *S*-sulfuration on C61 only (**Figure 5** and **Supplementary Table S1**). However, the biological implications of this modification are not yet known.

## Conclusion

In this study, we investigate the functions of three of the four Trx-like proteins in *S. aureus* strain Newman using an *in vivo* mechanism-based profiling strategy to trap candidate cellular substrates of TrxA, TrxP, and TrxQ. Each of these three Trx profiles are largely distinct from one another and feature non-overlapping sets of proteins save for a number of highly cell-abundant proteins involved in translation, as well as the major thiol peroxidase, Tpx. We then validate these findings using persulfidated pyruvate kinase, PykA-SSH, as a model substrate, and confirm that persulfide reduction by a Trx occurs through a mixed disulfide intermediate between the formerly persulfidated thiol and the attacking cysteine of the Trx, analogous to what occurs in disulfide reduction by TrxA (**Figure 1**), but with the release of H_2_S (Peng et al., 2017b).

PykA was identified in the Trx profiles of TrxP (at C8, C144, and C410), and *S*-sulfuration of mammalian PK has been previously demonstrated (Longen et al., 2016). TrxP may well function as a major depersulfidase in *S. aureus* at least under conditions of sulfide or RSS misregulation given its wider reach into bacterial metabolism relative to TrxA and TrxQ under these conditions. However, the canonical thioredoxin TrxA may also contribute significantly to this process under ambient growth conditions, given the higher catalytic efficiency of TrxA relative to TrxP in PykA persulfide reduction. Although it is of interest that the same three Cys residues, C8, C144, and C410, are *S*-sulfurated in cells and form intermolecular crosslinks specifically with TrxP, the *in vitro* biochemical data are not consistent with this apparent cellular specificity, since TrxA has high persulfide reduction activity toward PykA-SSH (**Figure 4**). This suggests that other cellular factors, e.g., relative protein concentrations, may augment what may be intrinsically small differences in substrate specificity *in vivo*.

The origin of the apparent cellular substrate specificity derived from the thioredoxin profiling experiments is not known, but local electrostatics may play a role. Indeed, previous studies reveal that electrostatic complementarity improves recognition and optimal orientation of the bound substrate leading to efficient electron transfer and reduction (Cheng et al., 2011). In addition, Trx harboring a positively charged “patch” showed a significantly higher reactivity toward the negatively charged glutathione disulfide, GSSG (Cheng et al., 2011). The structures of *S. aureus* TrxA (Roos et al., 2007), TrxP and PykA (Zoraghi et al., 2011) are known, and one can readily generate a homology model of TrxQ (**Supplementary Figures S7, S8**). The overall electrostatic surface potentials of the dithiol sides of these three Trx structures or models reveal that TrxA and TrxP are more similar than different, except in the immediate vicinity of the active site, which presents a weakly acidic patch in TrxP relative to the basic patch in TrxA (**Supplementary Figure S7**). The surface potential around C266 is distinct from the surface potentials of the three TrxP-specific sites, in that it is more acidic relative to the basic potentials that surround C8, C144, and C410 of PykA, thus possibly consistent with a role for electrostatic complementarity in driving Trx specificity (**Supplementary Figure S8**). The TrxQ model, on the other hand, suggests a strongly positive electrostatic surface potential around both the dithiol active site and C61; this may enhance the rates of reaction with what are generally negatively charged RSS at C61 (**Figure 5**). Beyond favorable electrostatics, a favorable entropy change has been implicated in the ability of Trx to interact with a range of cellular protein substrates in order to reduce their disulfide bonds (Palde and Carroll, 2015).

Our studies of pyruvate kinase and other candidate substrates *in vitro* are interesting from another perspective. The crystallographic structures of *S. aureus* PykA in the presence and absence of a *bis-*indole lead compound that targets MRSA PykA, only C410 is significantly exposed to solvent in either form of the enzyme (Zoraghi et al., 2011) a presumed necessary precondition for *S*-sulfuration and subsequent TrxP-mediated reduction (**Supplementary Figures S4, S8**). And yet, C8 and C144 are *S*-sulfurated in cells, and all three Cys are *S*-sulfurated *in vitro* using an inorganic (Na_2_S_4_) polysulfide donor. The regulatory impact of these modifications, however, is not yet known, but it is interesting to note that these three cysteines are capable of forming a mixed disulfide with the metabolic thiol, coenzyme A, in cells under conditions of diamide-induced oxidative or disulfide stress (Tsuchiya et al., 2018). The potential connection of TrxQ to Fe-S biogenesis, on the other hand, is also interesting given that the conserved C302 of the SufB subunit of the SufBC_2_D complex is expected to be very near where the active site [2Fe-2S] center is assembled prior to transfer to a client protein (**Supplementary Figure S5**) (Hirabayashi et al., 2015). In any case, although the functional determinants of the TrxQ and TrxP active sites, and the metabolic impact and degree to which TrxP is part of a regulatory cascade in cells, are not yet fully understood, these studies expand the biological footprint of the global antioxidant thioredoxin system in bacteria.

## Author Contributions

HP conducted all biochemical assays. YZ and JT ran mass spectrometry samples and analyzed crosslink results. HP and DG wrote the manuscript.

## Conflict of Interest Statement

The authors declare that the research was conducted in the absence of any commercial or financial relationships that could be construed as a potential conflict of interest.
