# Supplementary material for: Thioredoxin Profiling of Multiple Thioredoxin-Like Proteins in Staphylococcus aureus
Source: Front Microbiol. 2018 Oct 15;9:2385. doi: 10.3389/fmicb.2018.02385 (PMC6196236; doi:10.3389/fmicb.2018.02385)
Supplement: Supplementary file 2 [file Data_Sheet_1.pdf]

Supplemental Material

Thioredoxin profiling of multiple thioredoxin-like proteins in *Staphylococcus aureus*

Hui Peng,[1,2] Yixiang Zhang,[1,3] Jonathan C. Trinidad,[1,3] and David P. Giedroc*,[1,4]

[1]Department of Chemistry, Indiana University, 800 E. Kirkwood Drive, Bloomington, IN 47405-7102, United States

[2]Graduate Program in Biochemistry, Indiana University, 212 S. Hawthorne Drive, Bloomington, IN 47405, United States

[3]Laboratory for Biological Mass Spectrometry, Department of Chemistry, Indiana University, Simon Hall 120B, 212 S. Hawthorne Drive, Bloomington, IN 47405, United States

[4]Department of Molecular and Cellular Biochemistry, Indiana University, 212 S. Hawthorne Drive, Bloomington, IN 47405, United States

This file contains Supplemental Tables S1-S2 and Supplemental Figures S1-S8.

Supplemental Tables

Supplemental Table 1 A table summarizing the crosslinks formed between C32S TrxA or C32S TrxP with cysteine residues of PykA *in vitro* (see attached excel file).

Supplemental Table 2 Summary of MALDI-TOF masses of TrxQ under different conditions.[a]

Condition	Monoisotopic mass		Peak area	peptide 52-62	% persulfide[b]
	Calculated	Observed			
TrxQ	1444.7	1444.7	14899.5	IDLNFYPQFC(CAM)K	0.0
$TrxQ + Na_2S_4$	1444.7	1444.7	1054.1	IDLNFYPQFC(CAM)K	37.4
	1476.7	1476.6	629.5	IDLNFYPQFC(**S**CAM)K	

[a]TrxQ, as isolated, no reaction; $TrxQ + Na_2S_4$, 20 µM TrxQ, 400 µM Na_2S_4, 1 h, ambient temperature. [b]Calculated from the relative peak areas of the +32 peptide and unmodified peptide peaks.

Supplemental Figures

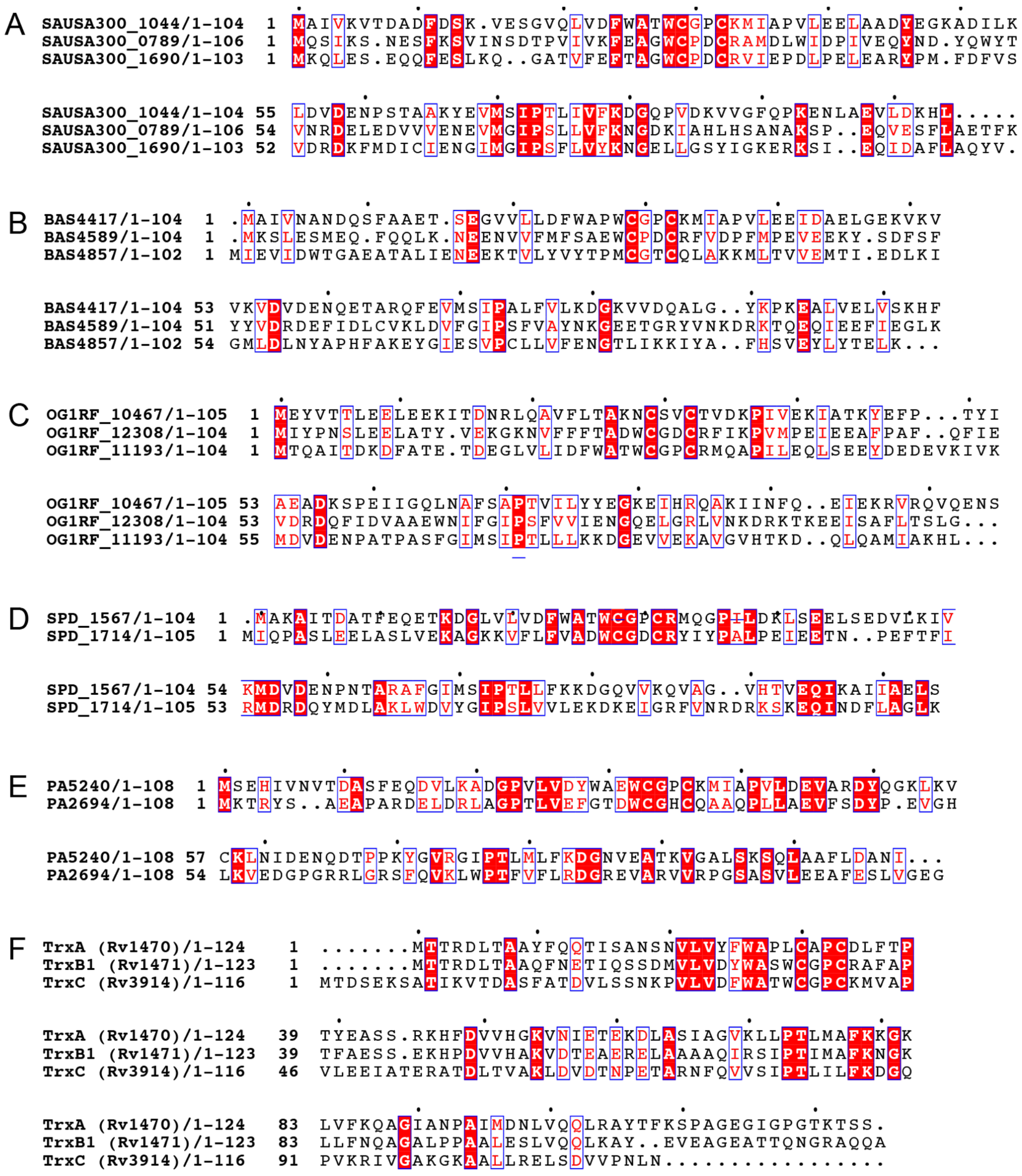

Supplemental Figure 1. Multiple sequence alignment of thioredoxin-like proteins from selected pathogenic bacteria, including A) *S. aureus* FPR3757 (USA300), B) *Bacillus anthracis* Sterne, C) *Enterococcus facaelis* OG1RF, D) *Streptococcus pneumoniae* D39, E) *Pseudomonas aeruginosa* PAO1 and F) *Mycobacterium tuberculosis* H37Rv. The Trx protein with W**C**<u>GP</u>**C** motif is presumed to be the authentic thioredoxin, often (but not always) denoted TrxA, with TrxP-like thioredoxins characterized by a W**C**<u>PD</u>**C**+ (+, K/R/H) active site motif (this work).

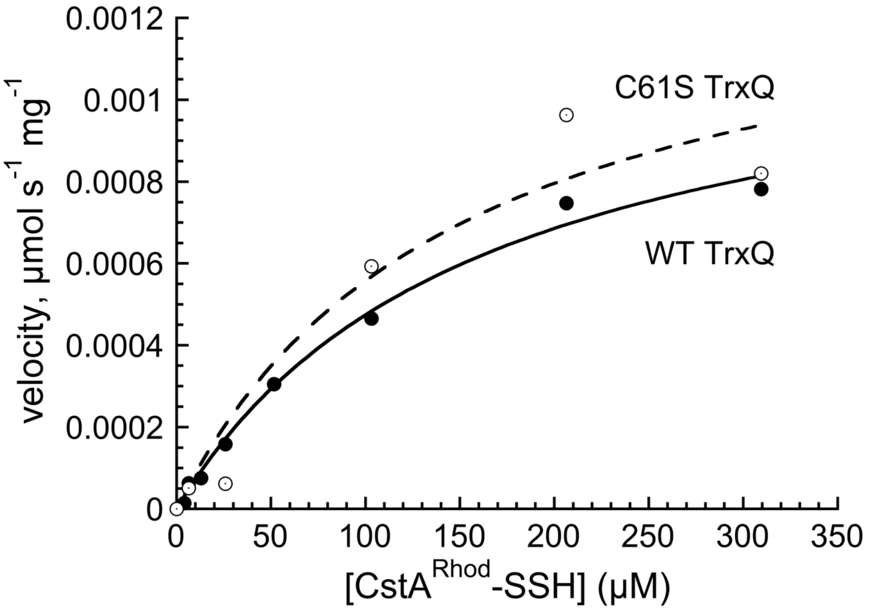

Supplemental Figure 2. The persulfide reduction activities of the wild-type and C61S TrxQ proteins toward *S*-sulfurated *S. aureus* CstA rhodanese domain ($CstA^{Rhod}$-SSH) as a substrate. The initial velocities of the enzyme as a function of $CstA^{Rhod}$-SSH concentration (Higgins *et al.*, 2015) were measured and fit to the Michaelis-Menten model defined by the continuous lines through the data. C61S and wild-type TrxQs show similar K_m (150 ± 113 and 160 ± 25 μM for wild-type and C61S TrxQ, respectively) and V_{max} (0.0012 ± 0.0001 and 0.0014 ± 0.0004 μmol s^{-1} mg^{-1} protein for wild-type and C61S TrxQ, respectively) under these conditions.

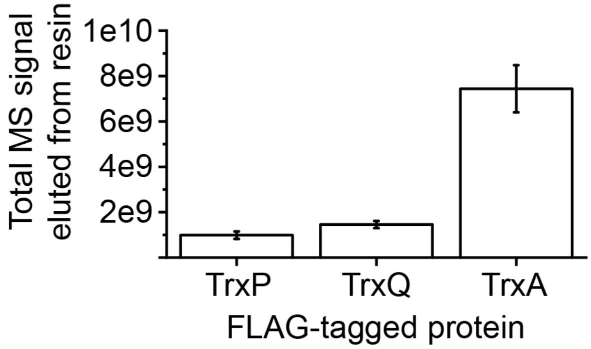

Supplementary Figure 3. Quantitation of the mass spectrometry (MS) signal intensity of all peptides derived from FLAG-tagged TrxP, TrxQ and TrxA eluted from the anti-FLAG resin and identified by LC-MS/MS. The bars shown represent the mean and the standard deviation from the three biological replicate experiments carried out in each case.

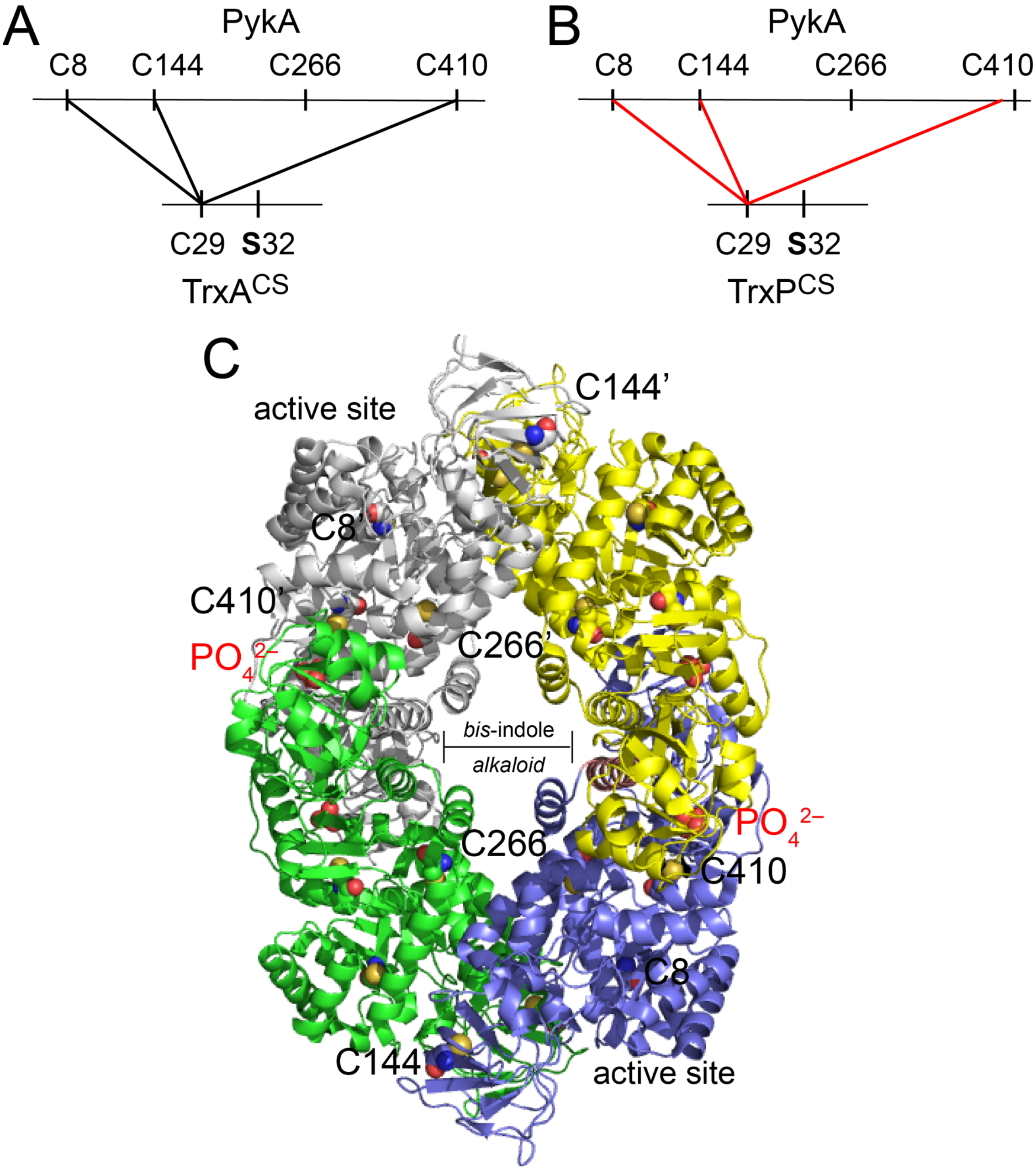

Supplemental Figure 4. *S*-sulfuration of *S. aureus* pyruvate kinase (PykA). Illustrations showing the crosslinks formed between the attacking cysteine of TrxA (A) or TrxP (B) with cysteine residues of PykA *in vitro*. TrxA and TrxP, of which the resolving cysteine is mutated to serine, are designated $TrxA^{CS}$ and $TrxP^{CS}$, respectively. The crosslinks which are also detected *in vivo* are highlighted in *red*. (C) Ribbon representation of the structure of “apo” pyruvate kinase from *S. aureus* strain MRSA252, with each subunit shaded differently (3T05) (Zoraghi et al., 2011). The four cysteines residues as well as bound phosphate anion are highlighted by CPK shading in spacefill, with the approximate positions of active site and *bis*-indole alkaloid binding site (a single molecule intercalates between the two helices positioned at each of the two small interfaces, one of which is shaded *salmon* on the *yellow* protomer) shown for reference.

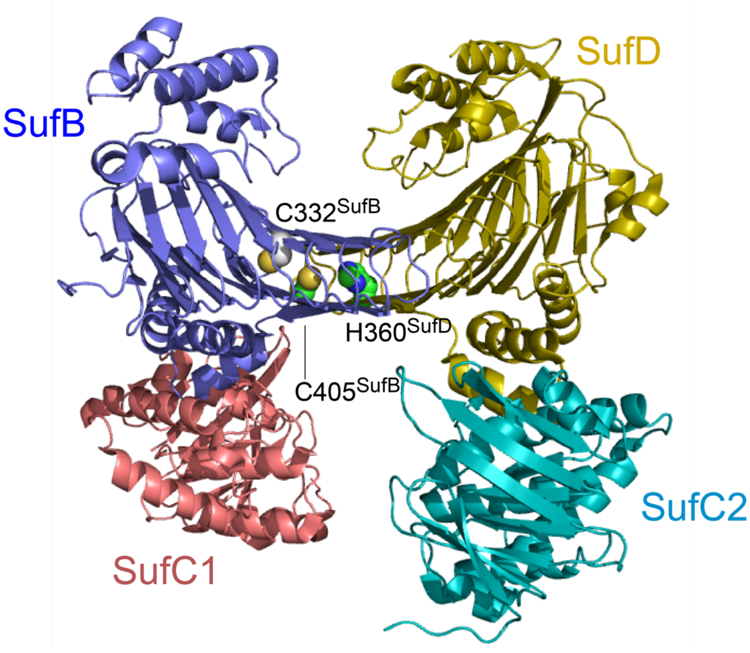

Supplementary Figure 5. Structural insights from a candidate TrxQ substrate protein. The structure of *E. coli* SufBC$_2$D complex (PDB code 5AWF) (Hirabayashi et al., 2015). C405^{SufB} and H360^{SufD} are required for Fe-S biogenesis and proposed to coordinate the [2Fe-2S] cluster that forms on this scaffold assembly. The conserved cysteine, C332^{SufB}, also highlighted in *sphere*, corresponds to C302 in the *S. aureus* SufB which is *S*-sulfurated in cells and is crosslinked to TrxQ in our profiling experiments (**Table 1**). The S$^{\gamma}$ atoms of C332 and C405 are just 14.4 Å apart in this inactive ATP-free conformation.

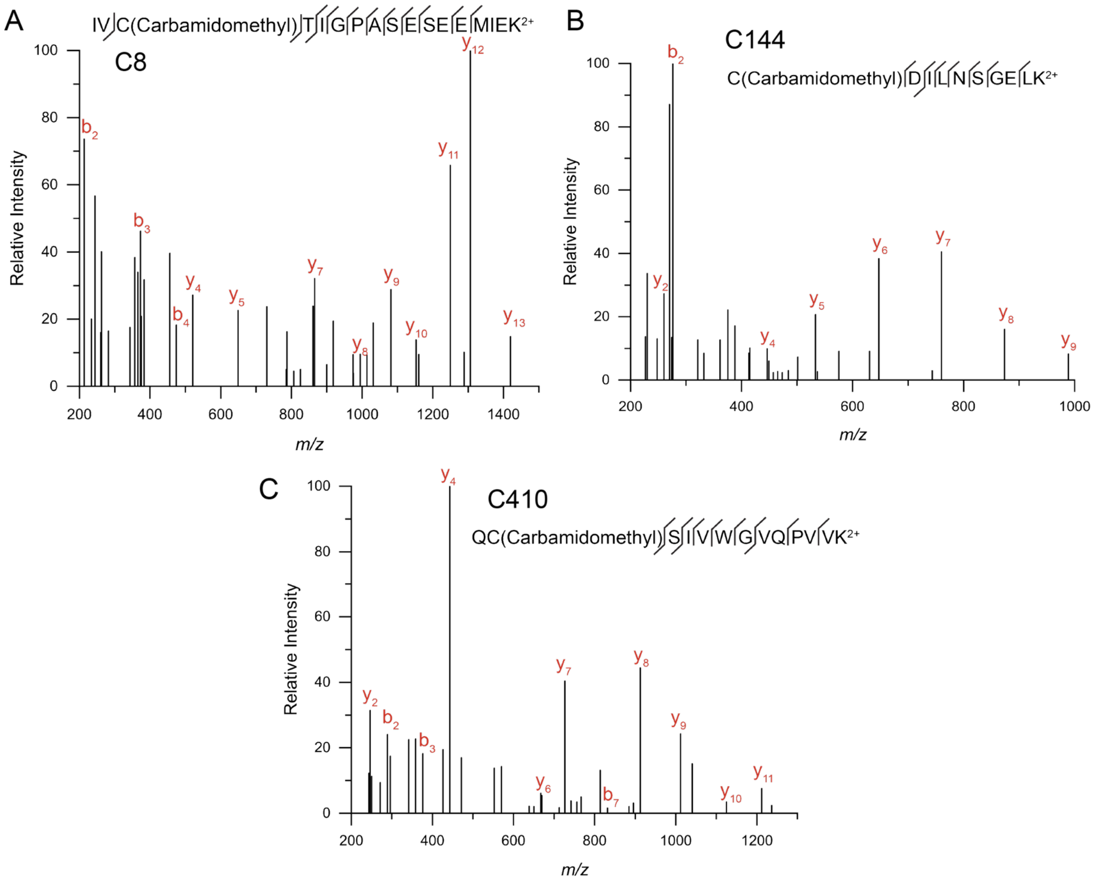

Supplemental Figure 6. MS/MS spectra reveal those cysteine residues of PykA as detected as *S*-sulfurated *in vitro*. Fully reduced PykA subjected to incubation with Na_2S_4 (PykASSH) was processed *in vitro* through the proteome *S*-sulfuration profiling workflow (Peng et al., 2017) in which *in vitro* *S*-sulfurated cysteine-containing peptides are specifically eluted and quantified as carbamidomethylated cysteine-containing peptides. Tandem mass spectra of peptides (+2 charge state) containing Cys8 (A), Cys144 (B) and Cys 410 (C) of PykA revealing that all three Cys persulfidated to some extent *in vitro*. Data were not obtained for the remaining C266-containing peptide in this experiment since the tryptic peptide is too short. See main text for additional details.

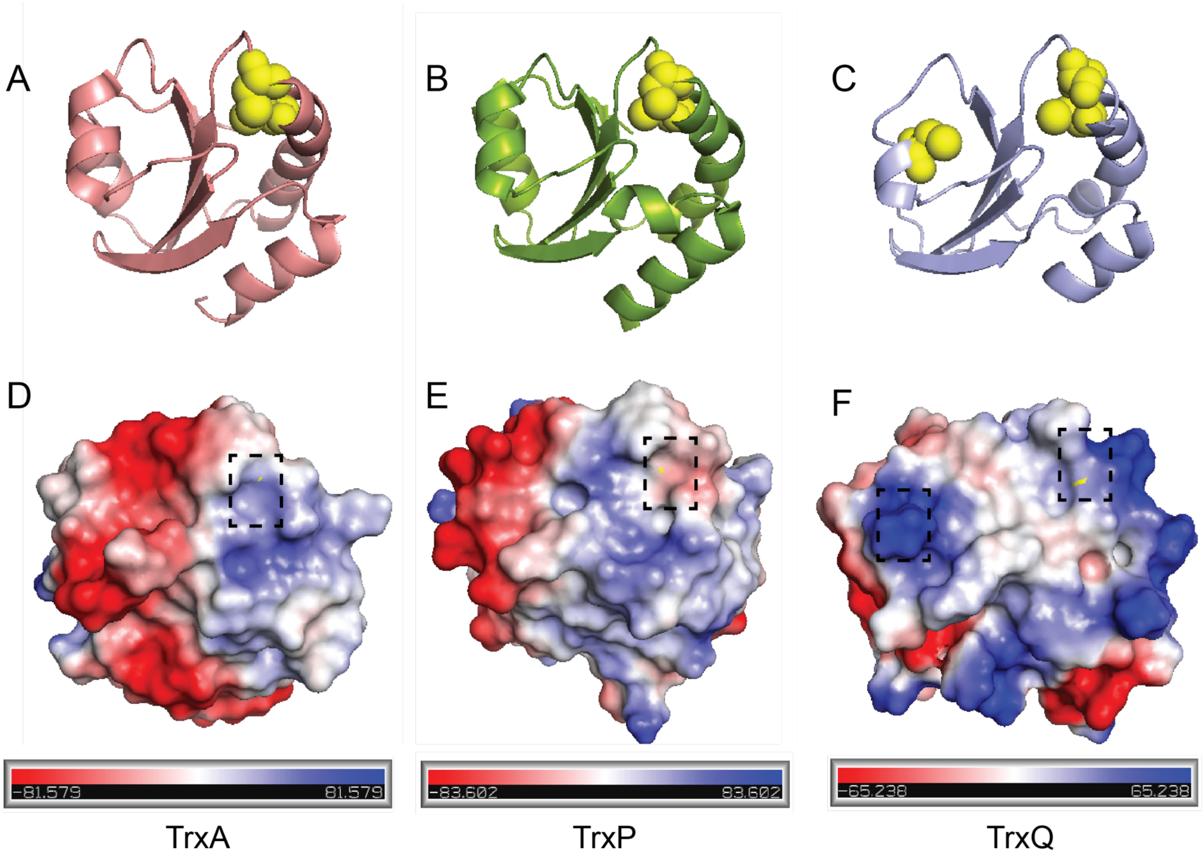

Supplemental Figure 7. Electrostatic surface potentials of TrxA, TrxP and TrxQ. Ribbon structures of TrxA (PDB entry 2O7K) and TrxP (4RUV) are shown in (A) and (B) respectively. The structure of TrxQ is a homolog model based on the structure of TrxP, in (C). *Yellow* spheres show cysteine residues in space-fill representation. The electrostatic surfaces that derive from these models of TrxA, TrxP and TrxQ are show in (D), (E) and (F) respectively. The dashed rectangles highlight the approximate positions of the cysteine residues in each model.

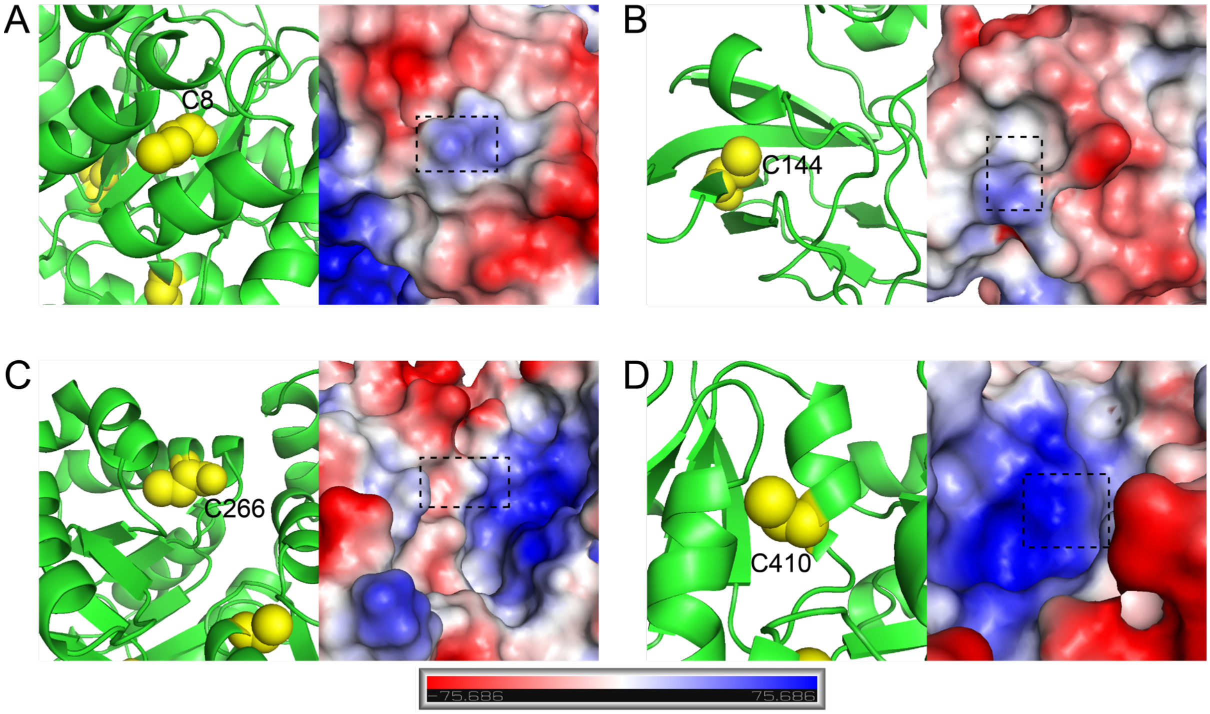

Supplemental Figure 8. Electrostatic surface of cysteine residues of *S. aureus* MRSA252 PykA (PDB entry 3T05). The local ribbon structure and electrostatic surface potentials around each of the four cysteine residues in PykA (Zoraghi et al., 2011), Cys8 (A), Cys144 (B), Cys266 (C), Cys410 (D). Cysteine residues are shown in *yellow* space-fill representation (*left*) with their corresponding electrostatic surface potentials highlighted by dashed rectangle (*right*). Positive charge is shown in *blue* and negative charge is shown in *red*. The solvent accessibility of the S^{γ} atoms of each Cys is 0 $Å^2$, 0 $Å^2$, 0 $Å^2$, and 15.2 $Å^2$, for cysteine residues 8, 144, 266 and 410, respectively, revealing that in this "apo" state in which only PO_4^{2-} occupies the effector sites, C410 S^{γ} is the only (partially) solvent-exposed Cys. In the related structure of the *bis*-indole alkaloid complex (PDB entry 3T07), only C410 S^{γ} is also partially exposed to solvent (9.5 $Å^2$) (Zoraghi et al., 2011).

References

Higgins, K.A., Peng, H., Luebke, J.L., Chang, F.M., and Giedroc, D.P. (2015). Conformational analysis and chemical reactivity of the multidomain sulfurtransferase, *Staphylococcus aureus* CstA. *Biochemistry* 54(14)**,** 2385-2398. doi: 10.1021/acs.biochem.5b00056.

Hirabayashi, K., Yuda, E., Tanaka, N., Katayama, S., Iwasaki, K., Matsumoto, T., et al. (2015). Functional dynamics revealed by the structure of the SufBCD complex, a novel ATP-binding cassette (ABC) protein that serves as a scaffold for iron-sulfur cluster biogenesis. *J Biol Chem* 290(50)**,** 29717-29731. doi: 10.1074/jbc.M115.680934.

Mochalkin, I., Miller, J.R., Evdokimov, A., Lightle, S., Yan, C., Stover, C.K., et al. (2008). Structural evidence for substrate-induced synergism and half-sites reactivity in biotin

carboxylase. *Protein Sci* 17(10), 1706-1718. doi: 10.1110/ps.035584.108.
Peng, H., Zhang, Y., Palmer, L.D., Kehl-Fie, T.E., Skaar, E.P., Trinidad, J.C., et al. (2017). Hydrogen sulfide and reactive sulfur species impact proteome *S*-sulfhydration and global virulence regulation in *Staphylococcus aureus. ACS Infect Dis* 3(10), 744-755. doi: 10.1021/acsinfecdis.7b00090.
Zoraghi, R., Worrall, L., See, R.H., Strangman, W., Popplewell, W.L., Gong, H., et al. (2011). Methicillin-resistant *Staphylococcus aureus* (MRSA) pyruvate kinase as a target for bis-indole alkaloids with antibacterial activities. *J Biol Chem* 286(52), 44716-44725. doi: 10.1074/jbc.M111.289033.
